# Effects of Dietary Indole-3-Acetate Sodium on Laying Performance, Egg Quality, Serum Hormone Levels and Biochemical Parameters of Danzhou Chickens

**DOI:** 10.3390/ani11030619

**Published:** 2021-02-26

**Authors:** Linlin Chen, Jun Xia, Lei Wang, Zhaobin Wang, Qi Mou, Yan Zhong, Yali Li, Qiye Wang, Jing Huang, Pengfei Huang, Huansheng Yang

**Affiliations:** Hunan Provincial Key Laboratory of Animal Intestinal Function and Regulation, College of Life Sciences, Hunan Normal University, Changsha, Hunan 410081, China; lin-linchen@foxmail.com (L.C.); xiajun913@foxmail.com (J.X.); wanglei_kdq@126.com (L.W.); anywang0914@yahoo.com (Z.W.); mou57@foxmail.com (Q.M.); zhongyanzyzy@foxmail.com (Y.Z.); yalili@hunnu.edu.cn (Y.L.); wangqiye@hunnu.edu.cn (Q.W.); jinghuang@foxmail.com (J.H.)

**Keywords:** indole-3-acetate sodium, laying performance, serum hormone levels, serum biochemical parameters, Danzhou chickens

## Abstract

**Simple Summary:**

The laying performance of hens plays a major role in the production of laying hens because it determines the economic benefits of farmers. Indole-3-acetate sodium is a derivative of indole-3-acetate (a major plant growth hormone). This study shows that indole-3-acetate sodium inclusion in diets improved the serum reproductive hormone levels and egg production performance of Danzhou chickens. We found that indole-3-acetate sodium could be considered as an effective feed additive to improve the laying performance of hens, but more comprehensive studies need to be performed in the future.

**Abstract:**

This study was conducted to investigate the effects of indole-3-acetate sodium (IAA-Na) inclusion in diets on the egg production performance, egg quality, intestinal tissue morphology, serum hormone levels and biochemical parameters of Danzhou chickens to preliminarily explore the efficacy of IAA-Na as a feed additive. A total of 192 Danzhou chickens (50 weeks old) were randomly assigned to 2 groups of 96. The diets for the treatment group consisted of the basal diets, supplemented with IAA-Na (200 mg/kg). The formal feeding trial lasted for four weeks. The results showed that the feed supplemented with IAA-Na not only increased the laying rate (*p* < 0.05) and egg yolk ratio (0.05 < *p* < 0.1), but also significantly reduced the feed:egg ratio (*p* < 0.05). In addition, the dietary supplementation of IAA-Na significantly increased the serum estradiol levels (*p* < 0.05) and decreased serum alkaline phosphatase activity (*p* < 0.05). Compared with the control group, the addition of IAA-Na to the diet had no significant effect on the intestinal tissue morphology or serum antioxidant capacity of Danzhou chickens. This study preliminarily provides evidence that dietary IAA-Na can improve laying performance, indicating that IAA-Na is a potentially effective feed additive for laying hens, but further studies are required before arriving at definite conclusions.

## 1. Introduction

Eggs are an important source of nutrients for humans. Although egg production is one of the most efficient industries in animal production nowadays, there remains great potential for further advancement. Therefore, improving the health status of poultry, improving laying performance and prolonging the peak production period have become a research field of great concern. A large number of antibiotic growth promoters are being used on laying hens to reduce the occurrence of diseases and improve the performance of laying hens [1]. However, the extensive use of antibiotics in poultry farms is one of the biggest threats to global health and food security [2]. Therefore, the poultry industry has been looking for new feed additives to improve the feed efficiency, egg production performance and health of poultry.

The effects of plant hormones on various aspects of plant growth and development have been widely studied and commercialized. Previous studies have shown that plant hormones, such as gibberellin [3] and daidzein [4,5], have a positive effect on the laying performance of poultry. Indole-3-acetic acid (IAA) is a major plant growth hormone that affects cell expansion, division and differentiation [6,7,8], and animals can obtain IAA from the intestinal absorption of a vegetable-rich diet or from the synthesis of tryptophan in various tissues [9,10]. It has been reported that IAA and its different derivatives have anti-cancer properties [11,12,13]. Preceding studies with cells and mice have revealed the anti-oxidative and anti-inflammation effects of IAA [14,15,16]. Researchers investigated the subacute toxic effects of IAA by investigating the different hematological and biochemical profiles of mice injected with IAA, which gave valuable support to using this plant hormone safely for agricultural purposes [17]. However, so far, there have been few reports on the effects of IAA as a feed additive on livestock and poultry products. Therefore, it may be feasible to research and develop the indole-3-acetate sodium (IAA-Na) plant hormone as a potential feed additive to improve the laying performance of Danzhou chickens.

The aim of this study was to test the effects of dietary IAA-Na supplementation on the laying performance, egg quality, ovary tissue morphology, intestinal tissue morphology, serum biochemical parameters and hormone levels of Danzhou chickens, as well as provide a theoretical basis for developing IAA-Na as a potential feed additive.

## 2. Materials and Methods

All procedures and the use of animals in this experiment were carried out in accordance with the Hunan Normal University Animal Ethics Committee guidelines (AEC number 2019/230).

### 2.1. Animals and Experimental Treatments

A total of 192 Danzhou hens (50 weeks old) housed in wire cages were randomly allotted to 2 groups of 96. Each group was further divided into six replicate blocks of 8 cages with 2 hens per cages. The diets for the treatment group consisted of basal diets (Table 1) supplemented with IAA-Na (200 mg/kg), which was evenly mixed to make pelleted experimental diets. IAA-Na was provided by the Shanghai Yuanye Bio-Technology Co., Ltd. (Shanghai, China). During the entire 28 day trial period, all hens had free access to feed and water. All hens were housed under routine conditions for the temperature, humidity, illumination (6 h light and 8 h dark in a 24 h day) and ventilation.

### 2.2. Data Recording, Sample Collection and Analysis

The health condition and laying performance were assessed daily. At the end of the trial, a single healthy laying hen was randomly selected from each group replicate, with 6 in each group, for a total of 12 hens, which were then sacrificed. Blood samples were collected from the axillary vein into vacuum tubes containing coagulant. After centrifugation, serum was collected and stored at −80 °C. The intestinal samples were fixed with a 4% formaldehyde-phosphate buffer and stored at room temperature for histological examination. Specimens of cross-sections of the intestinal segments were embedded in paraffin wax. The samples were then sectioned to a thickness of 5 mm and stained with hematoxylin and eosin. All tissue sections were determined under a microscope, using an image processing and analysis system (Leica Imaging Systems Ltd., Cambridge, UK). The villus height (VH), crypt depth (CD) and villus width (VW) of each intestine were measured by Program Image-pro Plus 6.0.

### 2.3. Laying Performance and Egg Quality

Egg production as well as the egg mass and feed intake were recorded to calculate the average total egg weight, average laying rate, average daily feed intake (ADFI) and feed:egg ratio (grams of feed consumed per gram of egg) during the first two weeks after the formal experiment. At the end of the second week of the trial period, two eggs were randomly selected from each repetition to evaluate the egg quality and cholesterol content. The cholesterol content of the egg yolk was determined using a kit from the Nanjing Jiancheng Institute of Biological Engineering.

### 2.4. Serum Biochemical, Hormone and Antioxidative Stress Analysis

An automatic biochemical analyzer was used to detect the serum biochemical indicators of the laying hens, including the total cholesterol (TC), high-density lipoprotein (HDL), low-density lipoprotein (LDL), total protein (TP), albumin (ALB), alanine aminotransferase (ALT), as well as the aspartate aminotransferase (AST) and alkaline phosphatase (ALP) activity and the triglyceride (TG), glucose (Glu), calcium (Ca) and urea nitrogen (BUN) contents. The total antioxidant capacity (T-AOC), catalase activity (CAT), superoxide dismutase activity (SOD) and malondialdehyde (MDA) contents in the serum were assayed by using commercial kits (Jiancheng Bioengineering Institute, Nanjing, China). The concentrations of the serum hormone parameters, including estradiol (E2), follicle-stimulating hormone (FSH) and luteinizing hormone (LH), were also assayed using Nanjing Jiancheng Bioengineering Institute assay kits (Nanjing, China).

### 2.5. Statistical Analysis

Data analyses were performed by an independent-sample T-test using SPSS 20.0. Data were presented as means with a standard error (means ± SEM). Differences were considered as statically significant at *p* ≤ 0.05 and highly significant at *p* ≤ 0.01, unless otherwise stated.

## 3. Results and Discussion

### 3.1. Laying Performance

The laying performance of hens plays a major role in the economic benefits of farmers. As shown in Table 2, there was no apparent effect on the ADFI and average egg weights with dietary IAA-Na supplementation compared to the control (CON) group. However, dietary supplementation with IAA-Na not only significantly increased the laying rate (*p* < 0.05), but also significantly reduced the feed:egg ratio (*p* < 0.05).

### 3.2. Egg Quality

Eggs are an important source of nutrients for humans, and it is well known that their composition can be modified through the manipulation of laying hen diets [18]. As shown in Table 3, in this study, the inclusion of IAA-Na in the diet of laying hens had no significant effect on the values of the egg shape index, eggshell strength, eggshell thickness, eggshell quality, yolk index, Haugh unit, egg yolk color or cholesterol content. However, dietary supplementation of IAA-Na tended to significantly increase the yolk ratio of the eggs (0.05 < *p* < 0.1). The ratio of the egg yolk is known as an important indicator to measure the nutrition of eggs, and increasing the ratio of egg yolk is equivalent to increasing the overall nutritional level of eggs [19].

### 3.3. Serum Antioxidant Activity Analysis

Antioxidative stress is one of the important factors affecting the performance and egg quality of laying hens. As shown in Table 4, no changes in the serum T-AOC, CAT or SOD concentrations were observed in the experimental treatments compared with the control group. The MDA content in the IAA-NA group was slightly decreased, but the difference was not significant.

### 3.4. Serum Hormone Analysis

**Figure 1:**
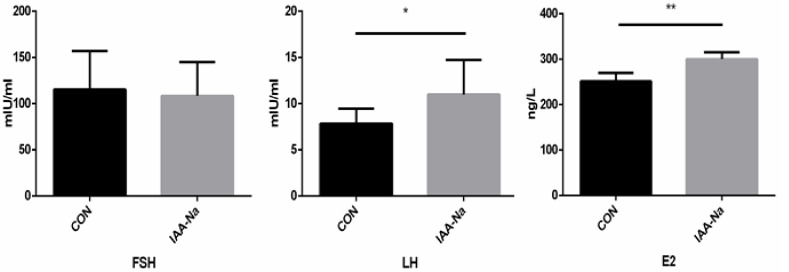
Effect of dietary IAA-Na supplementation on serum hormone levels in Danzhou chickens. Note: CON = basal diet, and IAA-Na = basal diet plus 200 mg/kg diet sodium indole-3-acetate. E2 = estradiol; FSH = follicle-stimulating hormone; and LH = luteinizing hormone. * and ** indicate a trend to a significant difference (0.05 ≤ *p* < 0.10) and a statistically significant difference (*p* < 0.05), respectively.

The serum hormone level has been considered a sensitive indicator of laying performance [20]. As shown in Figure 1, diets supplemented with IAA-Na had significantly increased E2 levels (*p* < 0.05) and trended to significantly increased LH levels (0.05 < *p* < 0.1). However, there was no significant difference in the serum FSH concentrations. The variation of laying rates was mainly determined by the hypothalamic–pituitary–gonadal axis [21,22]. Previous studies have also shown that a diet supplemented with octacosanol significantly improved egg production by increasing the serum hormone levels in laying hens [23]. The improvement of the LH and FSH levels could maintain more efficient ovulation of the ovary, thus increasing egg production, decreasing the feed conversion efficiency and lengthening the peak period of laying [24]. It was postulated that IAA-Na in diets could promote productive performance by releasing higher serum LH and E2 levels. However, the experimental period was relatively short, so further research is needed in the future.

### 3.5. Serum Biochemical Analysis

Biochemical indicators in the blood can be used to display the health status of hens. No significant differences in ALB, AST, TP, BUN, TG, CHOL, HDL, LDL, GLU, ALB or Ca were found among the treatment group and the control group (*p* > 0.05) in the Table 5. Compared with the control, the concentrations of serum ALP in the supplemental IAA-Na diet-fed hens were significantly decreased (*p* < 0.05). The relationship between serum ALP and egg production remains obscure. The opposite relationship between dietary Ca and serum ALP has been revealed [25]. The serum ALP activity of the lay hen decreased (385 U/L, 326 U/L and 283 U/L) linearly with an increasing Ca dietary concentration (3.20%, 3.70% and 4.20%), and the serum ALP activity was in the range of the reference values for laying hens [26]. The values of serum ALP activity (285.76 U/L) by IAA-Na supplementation observed in our study were decreased significantly (in the range of the reference values for laying hens). One possible explanation is that dietary IAA-Na reduces the activity of serum ALP by regulating the body’s absorption of calcium in the diet, thereby promoting egg production, but the concrete mechanism has yet to be further studied.

### 3.6. Intestinal Histological Analysis

The dietary IAA-Na had no significant effect on the intestinal tissue morphology and structure of Danzhou chickens (Table 6).

## 4. Conclusions

This study was conducted to investigate the effects of IAA-Na inclusion in diets on the egg production performance, egg quality, intestinal tissue morphology, serum hormone levels and biochemical parameters of Danzhou chickens. The results showed that the feed supplemented with IAA-Na had significant beneficial effects on the laying rate and the secretion of reproductive hormones. According to our findings, IAA-Na may be considered as a potential feed additive to improve the laying performance of hens. However, since this experiment is preliminary an exploration experiment with a relatively short experimental period, a longer experiment period and larger scale experiments need to be performed in the future.

## Figures and Tables

**Table 1:** Dietary composition and nutrient levels of the basal diet for the Danzhou chickens ^1^.

Item	Content	Items ^3^	Content
Ingredient	%	Nutrient levels	
Corn	64.46	ME/(MJ/kg)	11.30
Wheat bran	5.00	Crude protein (%)	15.00
Soybean meal	12.64	Lysine (%)	0.60
Rapeseed meal	5.00	Methionine (%)	0.27
Feather meal	1.50	Tryptophan (%)	0.17
CaHPO_4_	1.94	Methionine + cysteine (%)	0.59
Limestone	8.25	Total phosphorus (%)	0.65
NaCl	0.21	Available phosphorus (%)	0.46
Premix ^2^	1.00	Calcium (%)	3.40
Total	100		

^1^ Values are expressed on an air-dried basis. ^2^ The premix provided the following per kg of diets: 80.0 × 104 IU VA; 25 × 104 IU VD3; 1100 IU vitamin E; 500 mg vitamin B2; 250 mg vitamin K3; 110 mg vitamin B1; 350 mg vitamin B6; 1 mg vitamin B12; 65 mg folic acid; 2100 mg niacin; 18 mg D-biotin, 2300 mg pantothenic acid; 1500 mg Cu; 5000 mg Fe; 4800 mg Mn; 4200 mg Zn; 23 mg selenium; and 40 mg iodine. ^3^ These values are calculated values.

**Table 2:** Effect of dietary indole-3-acetate sodium (IAA-Na) supplementation on the laying performance in Danzhou chickens.

Item ^1^	CON	IAA-Na	*p* Value
Initial laying rate (%)	36 ± 3	37 ± 2	0.788
Wk52			
Average egg weights (g/egg)	39.33 ± 0.49	39.67 ± 0.33	0.588
Laying rate (%)	36 ± 3	49 ± 2	0.007
Wk53			
Average egg weights (g/egg)	39.81 ± 0.46	40.50 ± 0.42	0.854
Laying rate (%)	36 ± 4	48 ± 3	0.035
Wk52-Wk53			
Average egg weights (g/egg)	39.58 ± 0.41	40.10 ± 0.35	0.337
Laying rate (%)	36 ± 4	48 ± 2	0.012
Feed:egg ratio	4.33 ± 0.33	3.40 ± 0.20	0.037
ADFI (g/d)	63.50 ± 1.26	63.33 ± 1.97	0.549

Note: CON = basal diet, and IAA-Na = basal diet plus 200 mg/kg diet sodium indole-3-acetate. ^1^ ADFI = average daily feed intake. Feed: egg ratio = (g of feed)/(g of egg). Average egg weight = (total weight of eggs)/(number of eggs). Laying rate = (number of eggs)/(number of hens). Values were considered statistically significant at *p* < 0.05.

**Table 3:** Effect of dietary IAA-Na supplementation on the egg quality in Danzhou chickens.

Item	CON	IAA-Na	*p* Value
Egg shape index	1.32 ± 0.01	1.32 ± 0.01	0.790
Egg shell strength (kg/cm^2^)	3.28 ± 0.21	3.04 ± 0.22	0.440
Eggshell thickness (mm)	0.30 ± 0.01	0.27 ± 0.01	0.141
Eggshell quality (g)	3.60 ± 0.12	3.28 ± 0.19	0.160
Yolk percentage	0.34 ± 0.01	0.41 ± 0.03	0.062
Yolk index	0.31 ± 0.01	0.32 ± 0.01	0.818
Haugh unit	56.98 ± 2.53	56.14 ± 2.37	0.810
Yolk color	6.90 ± 0.35	6.62 ± 0.36	0.608
Cholesterol content of yolk (mg/g)	12.08 ± 1.15	14.06 ± 0.54	0.125
Cholesterol content of whole egg (mg/g)	5.04 ± 0.23	4.72 ± 0.56	0.609

Note: CON = basal diet, and IAA-Na = basal diet plus 200 mg/kg diet sodium indole-3-acetate. Values were considered statistically significant at *p* < 0.05 and trended toward significance when 0.05 ≤ *p* < 0.10.

**Table 4:** Effect of dietary IAA-Na supplementation on serum antioxidant activity in Danzhou chickens.

Item ^1^	CON	IAA-Na	*p* Value
T-AOC (mM)	2.00 ± 0.11	1.89 ± 0.12	0.549
CAT (U/mL)	3.24 ± 1.43	4.50 ± 1.47	0.562
SOD (U/mL)	100.17 ± 2.93	99.17 ± 1.73	0.777
MDA (nmol/mL)	10.79 ± 3.58	4.94 ± 1.34	0.174

Note: CON = basal diet, and IAA-Na = basal diet plus 200 mg/kg diet sodium indole-3-acetate. ^1^ T-AOC = total antioxidant capacity; CAT = catalase activity; SOD = superoxide dismutase activity; and MDA = malondialdehyde. Values were considered statistically significant at *p* < 0.05.

**Table 5:** Effects of dietary IAA-Na supplementation on serum biochemical levels in Danzhou chickens.

Item ^1^	CON	IAA-Na	*p* Value
ALT (U/L)	1.70 ± 0.84	3.55 ± 0.89	0.173
AST (U/L)	261.00 ± 24.45	245.67 ± 9.20	0.544
TP (g/L)	44.00 ± 3.64	49.10 ± 1.80	0.217
BUN (mmol/L)	0.64 ± 0.27	1.08 ± 0.30	0.311
TG (mmol/L)	15.28 ± 1.79	16.12 ± 1.97	0.762
CHOL (mmol/L)	3.62 ± 0.35	3.91 ± 0.27	0.534
HDL (mmol/L)	0.88 ± 0.10	1.15 ± 0.16	0.219
LDL (mmol/L)	1.13 ± 0.13	1.09 ± 0.11	0.799
GLU (mmol/L)	14.53 ± 0.88	14.13 ± 0.16	0.702
ALP (U/L)	442.20 ± 49.52	285.67 ± 31.08	0.022
ALB (g/L)	26.64 ± 1.52	26.05 ± 0.52	0.372
Ca (mmol/L)	2.23 ± 0.30	2.21 ± 0.15	0.953

Note: CON = basal diet, and IAA-Na = basal diet plus 200 mg/kg diet sodium indole-3-acetate. ^1^ ALT = alanine aminotransferase; AST = aspartate aminotransferase; TP = total protein; BUN = blood urea nitrogen; TG = triglycerides; TC = total cholesterol; HDL = high-density lipoprotein; LDL = low-density lipoprotein; Glu = glucose; ALP = alkaline phosphatase; ALB = albumin; and Ca = calcium. Values were considered statistically significant at *p* < 0.05.

**Table 6:** Effects of dietary IAA-Na supplementation on the intestinal morphology and structure in Danzhou chickens.

Item ^1^	CON	IAA-Na	*p* Value
Duodenum			
VH (μm)	1532.13 ± 64.09	1669.36 ± 104.35	0.289
CD (μm)	277.39 ± 39.34	275.90 ± 9.80	0.971
VW (μm)	295.32 ± 28.76	291.94 ± 21.18	0.927
VH/CD	6.05 ± 0.80	6.08 ± 0.39	0.971
Jejunum			
VH (μm)	1462.88 ± 55.25	1461.51 ± 96.16	0.989
CD (μm)	273.80 ± 45.25	222.59 ± 7.20	0.337
VW (μm)	213.87 ± 18.65	213.32 ± 19.82	0.984
VH/CD	5.42 ± 0.78	6.44 ± 0.56	0.319
Ileum			
VH (μm)	976.02 ± 61.02	933.42± 124.64	0.760
CD (μm)	153.03 ± 16.65	160.48 ± 19.56	0.802
VW (μm)	227.78 ± 11.41	205.14 ± 13.86	0.236
VH/CD	6.77 ±0.89	5.83 ± 0.43	0.365
Cecum			
VH (μm)	150.52 ± 6.97	163.74 ± 5.73	0.174
CD (μm)	120.56 ± 10.24	138.91 ± 11.27	0.256
VW (μm)	103.69 ± 7.04	92.25.32 ± 3.78	0.179
VH/CD	1.30 ± 0.14	1.20 ± 0.63	0.534

Note: CON = basal diet, and IAA-Na = basal diet plus 200 mg/kg diet sodium indole-3-acetate. ^1^ VH = villus height; CD = crypt depth; VW = villus width; and VH/CD = villus height/crypt depth. Values were considered statistically significant at *p* < 0.05.

## Data Availability

Data supporting this study’s findings are available by fair request from the corresponding author.
